# Graphene Oxide-Assisted Morphology and Structure of Electrodeposited ZnO Nanostructures

**DOI:** 10.3390/ma13020365

**Published:** 2020-01-13

**Authors:** N. Ma. Rosas-Laverde, A. Pruna, D. Busquets-Mataix, D. Pullini

**Affiliations:** 1 Department of Materials and Mechanical Engineering, Universitat Politècnica de València, 46022 Valencia, Spain; tabata8404@yahoo.com; 2 Department of Materials, Escuela Politécnica Nacional, Quito 170571, Ecuador; 3 Center for Surface Science and Nanotechnology, Polytechnic University of Bucharest, 060042 Bucharest, Romania; 4 Institute of Materials Technology, Universitat Politècnica de València, 46022 Valencia, Spain; dbusquets@mcm.upv.es; 5 Gemmate Technologies, 10043 Torino, Italy; 6 Centro Richerche Fiat, 10043 Torino, Italy; daniele.pullini@crf.it

**Keywords:** ZnO, graphene oxide, electrodeposition, structure-directing agent

## Abstract

In this paper, ZnO electrodeposition was studied with the presence of graphene oxide (GO) exploited as a possible structure-directing agent. The effect of deposition potential and duration on the morphology and structure of ZnO was analyzed. The morphology and structure of the hybrids was analyzed by Raman spectroscopy, X-ray diffraction (XRD), and Scanning Electron Microscopy (SEM). The Raman results indicate a successful modification of ZnO with GO sheets and a hybridization threshold of 10 mg L^−1^ by the evolution of the defect related band of ZnO at 580 cm^−1^. The morphology results show that a low GO content only slightly influences the morphology and orientation of ZnO nanostructures while a high content as 10 mg L^−1^ changes the morphology in nanoplates and growth orientation to lateral. The results show that while GO participated in the deposition reaction, it has a two-fold role, also by structure-controlling ZnO, indicating that the approach is valid for the use of GO as a structure-directing agent for the fabrication of ZnO nanostructures by electrodeposition with varying morphologies and orientations.

## 1. Introduction

Zinc oxide (ZnO) is an n-type semiconductor highly employed in different devices, such as sensors, biosensor, solar cells, supercapacitor and catalysis fields [1,2,3,4] thanks to its interesting properties, including its wide band gap of 3.37 eV [4], resistivity control, high electrochemical stability, good electron transfer features and transparency in the visible wavelength region [1] in addition to being an abundant non-toxic and low-cost material.

It is well known that properties of materials at nano-scale are markedly dependent on their size, shape or morphology; thus, the control of features such as porosity, surface area or specific orientation has attracted much interest for improving the performance of ZnO-based devices [5]. In this respect, ZnO morphology is highly versatile as it encompasses nanorods, nanowires, nanotubes, nanowalls, nanocups nanobelts, nanorings, nanosprings, nanobowls, nanoflowers, nanohelices and nanoparticles [1,6,7,8]. For example, Wang et al. reported a novel and improved ethanol gas sensor based on electrodeposited flower-like ZnO microstructures [9], Psychoyios et al. fabricated a ZnO-based potentiometric cholesterol biosensor with improved adsorption capability by improving the surface area to volume ratio of ZnO structures [5], while Marimuthu et al. demonstrated that the efficiency of ZnO dye sensitized solar cells (DSSCs) was markedly improved for ZnO nanowalls [10].

A large range of techniques such as magnetron sputtering, spray pyrolysis, electrodeposition, sol-gel, chemical bath deposition, thermal methods pulsed laser ablation in liquid or gas environment or chemical vapor deposition have been applied for the synthesis of ZnO nanostructures and its composites [11,12,13,14,15,16]. Amongst these techniques, electrodeposition represents a great alternative as it provides excellent coating of varying geometries of substrates, it allows the control of morphology, thickness and crystallite size and aspect ratio of the deposit by simply varying the electrochemical parameters, including precursor concentration, bath temperature, deposition time or deposition potential/current [1,10] and it is a simple and cost- and time-efficient technique which does not require sophisticated experimental setups [1]. For instance, morphologies such as platelets, nanowalls and nanorods were reported by adjusting the electrodeposition potential and bath temperature [10]. Furthermore, the use of structure-directing agents during the electrodeposition has been applied to control the crystal orientation and thus the properties of ZnO materials [17]. For example, Eosin Y was reported to accelerate the growth process of ZnO and result in a porous film with a high surface area and enhanced electron transport that improved the ZnO-based dye sensitized solar cell´s efficiency [18,19,20]. Other examples of structure-directing agents include citric acid [20] and even a combination of agents such as Eosin Y and Eosin B that were applied to modify the porosity of ZnO to obtain an improved efficiency for dye-sensitized solar cells [21].

Lately, the use of graphene oxide as a structure-directing agent has attracted great research interest. Graphene, a two-dimensional carbon allotropic material shows a highly specific surface area (2630 m^2^ g^−1^), high mobility (15,000 m^2^ V^−1^ s^−1^) along with excellent electrical and mechanical properties thanks to which its composites exhibit improved performance [4,22,23,24]. Graphene oxide (GO), a derivative material of graphene [22] which can be obtained by simple wet chemistry methods [25], has decorative oxygen functional groups (e.g., hydroxyl, carboxyl and epoxy groups) on its surface and edges [26,27] which make it amphiphilic [28], a hydrophilic/hydrophobic structure, so that it can act as a surfactant and has the ability to control some characteristics of the final structure, including composition, structure and morphology [22,29]. The reports on GO as a shape-directing agent include carbon aerogels [30], bimetallic nanopowders [28], inorganic nanomaterials [29], heterogeneous 2D carbon nanostructures [31], carbon-based catalysts [32] or metal-organic frameworks (MOF) [33]. Zhanga et al. used GO to control the growth and induce the vertical orientation of magnesium molybdate nanosheets for anodes in a lithium battery [34]. With the help of GO as a structure-directing agent, the morphology can be tailored and result in an improved photocatalytic activity [33]. In other example, Lu et al. demonstrated that GO directly influences the growth kinetics and induces a concavity in the nanocubes of PtPd nanocrystals in its presence [35].

There is special interest in the synthesis of hybrid composites of ZnO nanocrystals with GO in order to obtain a synergistic effect towards enhanced performance [23] in sensing, energy storage, catalysis, photovoltaics and pollutant degradation [2,3,36,37,38]. It has been shown that GO could be applied as a scaffold in a ZnO/reduced GO hybrid (ZnO/rGO) [1] and contributes to enhanced performance thanks to attributes such as its high surface area [23]. The ZnO/rGO hybrids can be fabricated by different methods, including chemical vapor deposition, electrodeposition, hydrothermal deposition, spray hydrolysis or drop-casting/electrophoretic deposition [1,4,23,39]. Amongst said methods, the electrodeposition is a great alternative for the synthesis of ZnO/rGO hybrids as it allows for the control on the doping of ZnO nanostructures [40].

Herein, the synthesis of ZnO/rGO hybrids is reported by a one-step electrodeposition approach based on a simultaneous deposition of ZnO and electro-reduction of GO. The GO is shown to have a two-fold role of reagent and a structure-directing agent to control the growth of ZnO crystals. A systematic study on the effect of GO content and electrodeposition conditions such as potential and duration on the ZnO nanostructures is presented. The obtained results indicate that GO can be successfully applied to control the structure and orientation of ZnO electrodeposited nanostructures towards improving its performance in varying fields.

## 2. Materials and Methods

### 2.1. Materials

The chemicals were reagent grade (Alfa Aesar) and used as received. GO was obtained as presented previously [41]. All electrolytes were obtained with distilled water. Indium-doped tin oxide (ITO) coated conducting glass slides (~15 Ω/sq) were cleaned successively in soapy water, distilled water, acetone and isopropylic alcohol by ultrasonic treatment.

### 2.2. rGO-Assisted Electrodeposition of ZnO Nanostructured Films

The ZnO nanostructured films were synthesized on indium tin oxide (ITO)substrate from 0.1 M KCl supporting electrolyte containing 5 mM ZnCl_2_ and varying GO content up to 10 mg L^−1^ under continuous O_2_ flow. The deposition was performed by applying a constant potential ranging from −0.8 to −1 V for up to 600 s at 75 °C.

### 2.3. Characterization

The electrochemical deposition was performed using an Autolab potentiostat in a classical three-electrode electrochemical cell using ITO slides, Pt plate and saturated Ag/AgCl electrode as the working electrode, the counter one, and the reference electrode, respectively. X-Ray diffraction (XRD) spectra were acquired using a diffractometer (Bruker, D2 Phaser, Madrid, Spain) using the Cu Kα line of 1.54 Å. The morphology was analyzed with a scanning electron microscope (SEM, JSM-820 JEOL, Jeol, Tokyo, Japan) working at 20 kV. Raman spectra were recorded using a (inVia, Renishaw, Barcelona, Spain) microscope employing a 514-nm laser.

## 3. Results

**Figure 1:**
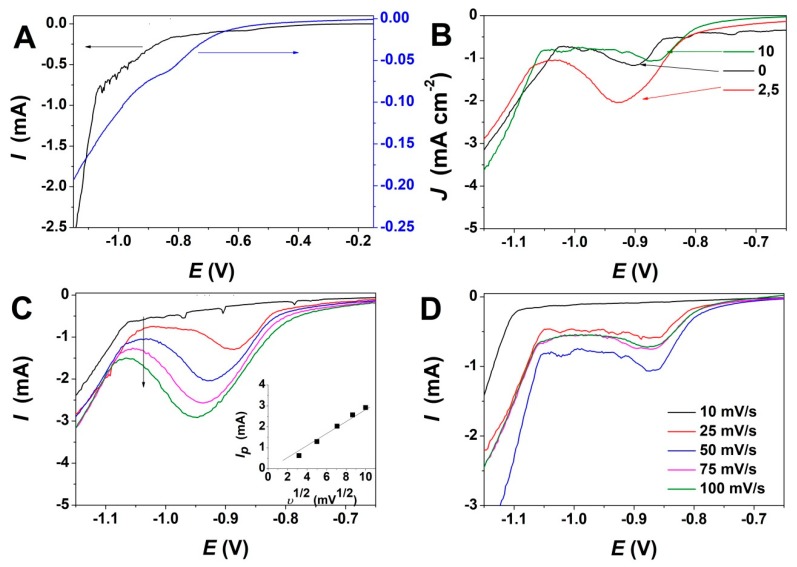
(**A**) Linear sweep voltammetry of ZnO electrodeposition in absence (left) and presence of GO (right) at 10 mV s^−1^; (**B**) Evolution of ZnO electrodeposition with content of GO (mg L^−1^) at a scan rate of 50 mV s^−1^; (**C**) Evolution of ZnO electrodeposition with the scan rate at a GO content of 2.5 mg L^−1^ (inset depicts the evolution of reduction peak intensity with the scan rate); (**D**) Evolution of ZnO electrodeposition with scan rate at a GO content of 10 mg L^−1^.

Linear sweep voltammetry (LSV) measurements were first performed in order to determine the evolution of reduction potential with GO addition. Figure 1 depicts the effect of GO content and varying scan rate on ZnO electrodeposition. As can be observed from the LSV curves in Figure 1A, a reduction peak appears at about −0.9 V in the absence of GO, while the addition of GO shifts the reduction peak to a more anodic value. The electrodeposition of ZnO takes place by a reaction mechanism based on the reduction of oxygen molecules and the formation of Zn(OH)_2_ which dehydrates at the applied temperature condition to form ZnO [11] and simultaneous GO reduction according to [42]:


GO + aH^+^ + be^−^ → rGO + cH_2_O (1)

Figure 1B shows the variation of the reduction peak for ZnO electrodeposition upon addition of varying GO content in the bath up to 10 mg L^−1^. For exemplification, the LSV curves recorded for 2.5 and 10 mg L^−1^ are presented only. At a low GO content, the reduction current increases and the peak potential is shifted to more cathodic values while in presence of higher GO content, the peak potential shifts back to its original position—see exemplification for GO to 10 mg L^−1^ where the LSV is similar to the one obtained in the absence of GO.

Figure 1C,D depicts the evolution of LSV curves for ZnO electrodeposition with a scan rate at a a GO content of 2.5 mg L^−1^ and 10 mg L^−1^, respectively. It is shown that upon low GO addition, the reduction potential is shifted negatively as the scan rate is increased. The deposition current increases, as indicated in the inset in Figure 1C. On the other hand, a GO content as high as 10 mg L^−1^ shows very little variation in the reduction potential and current for ZnO/rGO hybrid electrodeposition.

**Figure 2:**
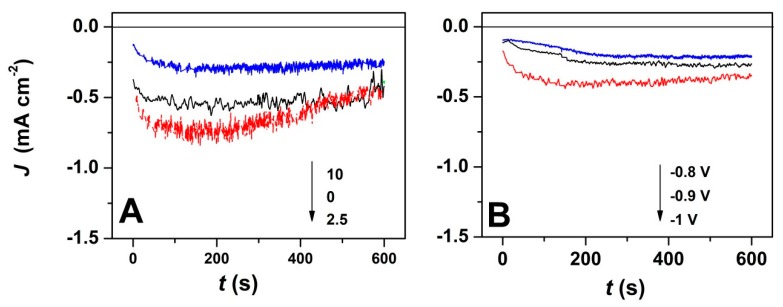
(**A**) Current transients for ZnO electrodeposition in presence of varying GO amount at an applied deposition potential of −1 V; (**B**) Current transients for ZnO electrodeposition with applied deposition potential in the presence of 7.5 mg L^−1^ GO.

Furthermore, the current transients corresponding to the potentiostatic electrodeposition of ZnO with GO addition and at varying deposition potentials were studied, as depicted in Figure 2. As it can be seen, the chronoamperometric curves exhibit a similar trend, independently of the electrodeposition conditions. The electrodeposition current decreases with the GO content, as shown in Figure 2A, while the nucleation process is completed in about 100 s. Nevertheless, the current increases with the applied deposition potential even in the presence of a high GO content (Figure 2B) which is expected to result in thicker deposit [43]. By increasing the applied potential, a steep current slope is observed, which results in the faster establishment of a current plateau (after about 100 s).

**Figure 3:**
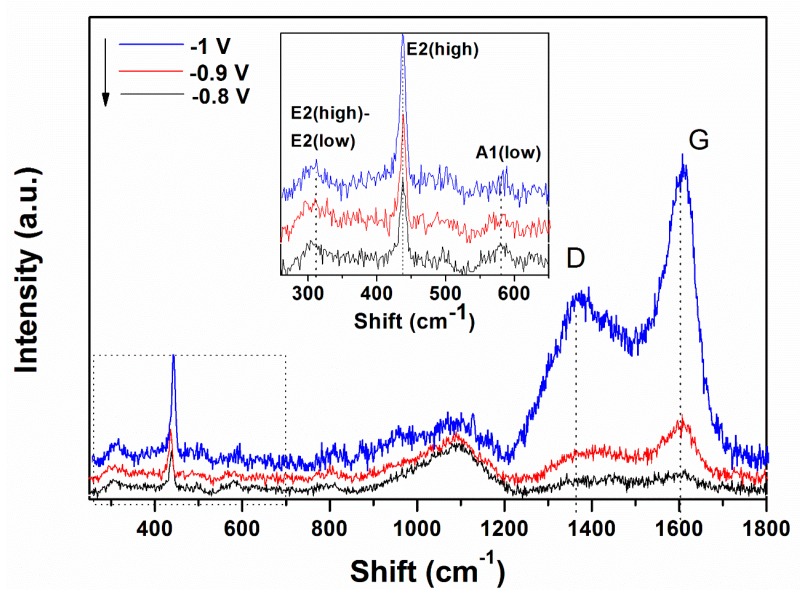
Raman spectra evolution of ZnO electrodeposited in presence of 10 mg L^−1^ GO as a function of the applied deposition potential.

Raman spectroscopy was carried out in order to characterize the ZnO/rGO hybrids. The formation of ZnO and the presence of rGO in the hybrid material obtained by a 1-step electrodeposition process is confirmed by the appearance of typical bands of both components, as shown in Figure 3. In the low wavenumber range, three bands located at 310, 440 and 580 cm^−1^ are attributed to ZnO [23,44].

The typical band of wurtzite ZnO at 440 cm^−1^ corresponding to non-polar optical phonon E_2H_ mode is observed to increase in intensity while the band located at 580 cm^−1^ associated with bulk defects, including oxygen vacancies, zinc interstitials or defect complexes comprising both of them [45] appears unaffected by the cathodic potential. On the other hand, in the higher wavenumber range, the bands at 1370 and 1602 cm^−1^ represent the typical D and G bands of GO which correspond to the disorder-induced mode in the hexagonal graphitic layer and sp^2^ carbons, respectively [44,46,47]. The intensity of D and G bands of GO increase with the applied deposition potential.

**Figure 4:**
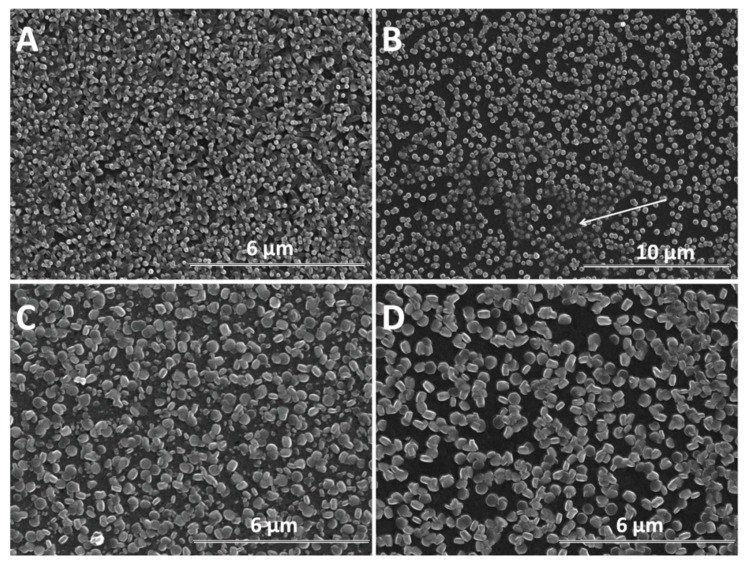
SEM images of ZnO electrodeposited as a function of GO content (mg L^−1^) at −1 V for 120 s: 0 (**A**), 2.5 (**B**), 7.5 (**C**), 10 (**D**).

SEM analysis was further performed in order to determine the morphological changes and growth directions of the ZnO nanostructures upon modification with GO as direct-structure agent. Given the importance of the deposition potential on the structure as well as the nucleation on the growth of ZnO, the combined effect of GO content with the electrodeposition potential and duration was analyzed. The result of the first stage of the deposition process −1 V for up to 120 s is presented in Figure 4. It can be seen that the density of ZnO nanostructures slightly diminished while their lateral size increases upon the addition of GO. The growth along the c-axis of ZnO nanostructures is observed to be affected at a higher GO content than 2.5 mg L^−1^. No significant change in the vertical orientation is observed at a low GO content (see GO sheets as indicated by the arrow in Figure 4B), while a high GO content results in the loss of preferred vertical orientation. The addition of GO appears to induce a rougher surface of ZnO [48] while the nanostructures change from nanorod to nanoplate morphology.

**Figure 5:**
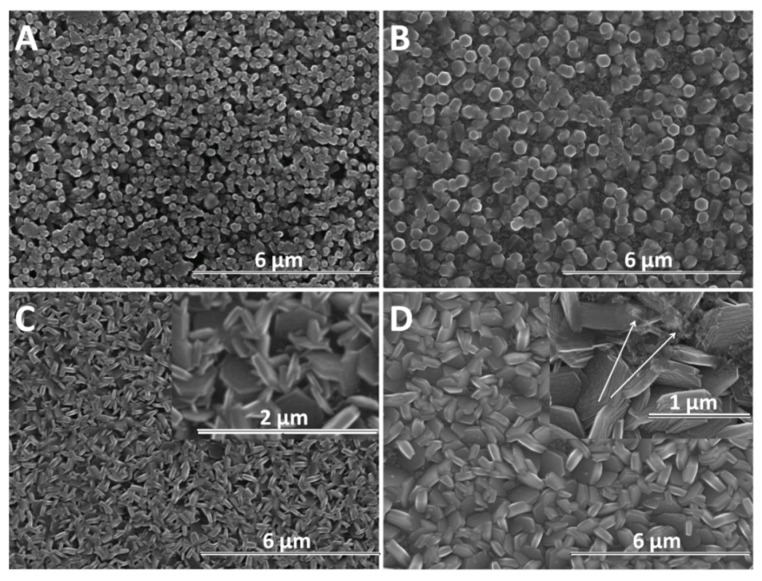
SEM images of ZnO electrodeposited as a function of GO content (mg L^−1^) at −1 V for 600 s: 0 (**A**), 2.5 (**B**), 7.5 (**C**), 10 (**D**).

Furthermore, the growth of nanostructures was studied with time, as presented in Figure 5. For exemple, the duration of 600 s is considered sufficient to evidence changes in the morphology of ZnO nanostructures due to the presence of GO. As expected, a larger diameter is observed in all cases, in agreement with Skompska et al. [49] and Arslan et al. [50]. The vertical growth of ZnO nanostructures is maintained at a low GO content up to 2.5 mg L^−1^ while a higher GO content shifts it to lateral and the morphology changes from nanorods to nanoplates, as shown for the shorter duration. The hybrids deposited at a high cathodic potential show thin GO sheets at the top of the ZnO nanostructures at a low GO content, as evidenced by the arrow in Figure 4B while at a higher content, the sheets are apparently missing. However, the images obtained for a longer deposition duration confirm the presence of agglomerated crumpled rGO sheets surrounding the ZnO nanostructures, as indicated by the arrows in Figure 5D.

**Figure 6:**
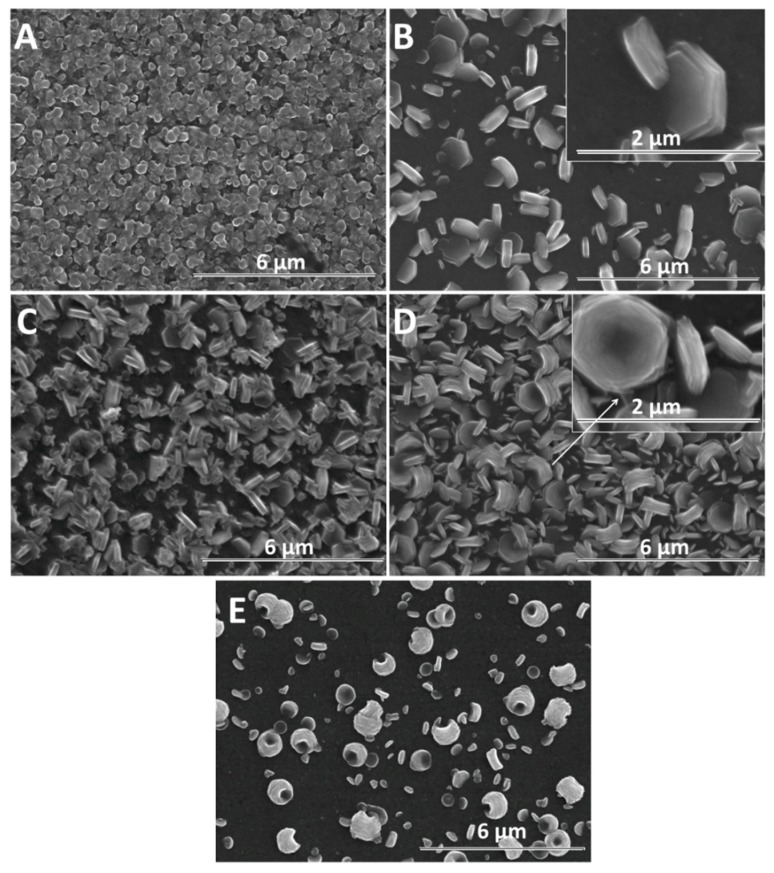
SEM images of ZnO electrodeposited as a function of GO content (mg L^−1^) at −0.8 V for 600 s: 0 (**A**), 2.5 (**B**), 7.5 (**C**), 10 (**D**). SEM image of ZnO electrodeposited in the presence of 10 mg L^−1^ GO at −0.7 V for 600 s (**E**).

Although a lower cathodic deposition potential is known to result in lower quality crystalline structures, the morphology of GO-modified ZnO nanostructures could be exploited to develop synergetic properties. Figure 6 shows the SEM evolution of ZnO nanostructures obtained at −0.8 V for 600 s with the GO content. Figure 6A shows a ZnO film constituted of dense nanorods without a clear definition of boundaries. As the ZnO nanostructures are not well defined at a lower deposition potential and the GO sheets are better allowed to migrate to the electrode, their effect on the morphology of ZnO at a high GO content is more evident. The morphology of ZnO changes from nanorods to nanoplates even at a low GO content—see Figure 6B and nanocontainers, upon increasing the GO content up to 10 mg L^−1^—see Figure 6D. At −0.8 V, all the films show a random orientation.

**Figure 7:**
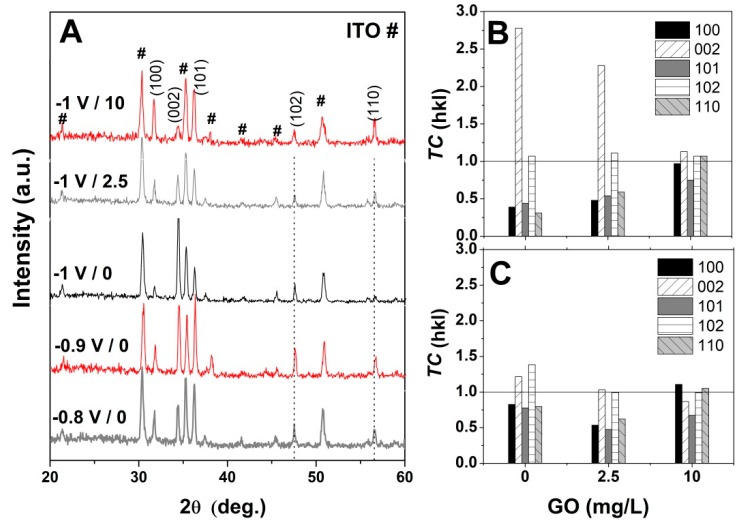
(**A**) XRD spectra evolution of non-modified ZnO with applied potential and GO content (mg L^−1^); (**B**) and (**C**) texture coefficient (*TC*) evolution of ZnO electrodeposited at −1 V and −0.8 V, respectively as a function of the GO content.

The influence of GO as a structure-directing agent for ZnO electrodeposition was analyzed by XRD. Figure 7A shows the evolution of XRD spectra of the pure ZnO with the applied deposition potentials in the absence of GO. As can be seen in Figure 7A, the typical peaks for the hexagonal wurtzite structure of ZnO (JCPDS card no. 36-1451) are identified beside the peaks corresponding to the substrate, that is, the diffraction peaks located at (100), (002), (101), (102) and (110) [37,51,52]. It can be observed that the preferred orientation towards c-axis growth increases with the cathodic potential, in agreement with the SEM images in Figure 5A and Figure 6A [51]. Upon the addition of GO, the ZnO/rGO hybrids presented similar diffraction peaks as the non-modified ZnO (not shown), in agreement with other reports [46].

Furthermore, the preferential growth was determined by calculating the texture coefficient *TC* by using the equation [49,50]: *TC* = (*I*_(*hkl*)_/*I_0_*_(*hkl*)_)/(*N*^−1^ *∑_n_ I*_(*hkl*)_/*I*_0(*hkl*)_), where *I* and *I_0_* are the measured relative intensity and standard intensity from the JCPDS file, respectively, for a plane (*hkl*), *N* is the reflection number in the difractogramm. A random orientation of the crystals is achieved when *TC ≈* 1 and a preferential one when *TC* > 1. Figure 7B,C show the texture coefficient (*TC*) evolution for ZnO electrodeposited in the presence of varying GO content and applied deposition potential. The obtained *TC* values indicate that the ZnO nanostructures obtained at −1 V exhibit a growth preference for (002) plane in the absence of GO [50] which it is maintained upon the addition of low GO content of 2.5 mg mL^−1^ while a high GO content of 10 mg L^−1^ results in the loss of preferential orientation, in agreement with Figure 4 and the XRD spectra in Figure 7. On the other hand, at lower cathodic deposition potential, the ZnO nanostructures do not show a preferential growth orientation. It can be observed that *TC* along (002) gradually decreases while that for other planes such as (100) and (110) increase with the GO content.

## 4. Discussion

The LSV curves in Figure 1A indicate that the presence of GO shifts the reduction potential, which is attributed to the reduction of GO sheets simultaneously with ZnO electrodeposition. The addition of low GO content to the electrolytic bath results in an increased reduction current, as can be observed in Figure 1B, which could be attributed to the GO acting as a scaffold and its decorating oxygen groups as a nucleation center for growing ZnO [37]. The reduction peak evolution with the scan rate depicted in Figure 1C indicates a linear increase in the deposition current along with a shift to more cathodic potential in the presence of low GO content, indicating an increasing deposition rate as the oxygen functional groups in GO are exposed and used as a scaffold to assist the growth of ZnO. On the other hand, a higher GO content in the electrolytic bath resulted in a negligible shift in the reduction peak current and potential with respect to the ZnO electrodeposition in the absence of GO, which is due to the increased density of GO sheets in the electrolytic bath that result in an agglomeration and thus, in the exposure of less oxygen groups that could be used for ZnO growth.

The chronoamperometric curves in Figure 2 show more stable deposition current at a GO content of 10 mg L^−1^ which is attributed to the homogeneous exposure of oxygen functional groups to easily react with Zn^2+^ ions due to denser GO sheets in the electrolytic bath [37]. The GO use as a direct-structure agent was reported to allow the synthesis of homogenous layers [35]. By employing a high GO content, the chrono-amperometric curves show a steady homogenous growth at −0.8 and −0.9 V, while at a higher cathodic potential of −1 V, a steep current slope is observed that is attributed to the faster formation of nuclei [2].

Raman spectroscopy is a very sensitive to the electronic structure. The typical Raman peak of wurtzite ZnO located at 440 cm^−1^ increases in intensity with the cathodic potential, indicating the improved crystallinity of ZnO in the ZnO/rGO hybrids with cathodic potential. The band associated with defects in ZnO (e.g., oxygen vacancies) at 580 cm^−1^ indicates the hybridization of ZnO with GO and shows that at a higher GO content (i.e., 10 mg L^−1^), the hybridization reaches a limitation as it did not change in location or full width half maximum. The peaks corresponding to GO indicate that the *I_D_/I_G_* ratio is relatively higher at −1.0 V than lower applied deposition potentials, which is attributed to an increased reduction degree [23,28,47,53,54,55].

As the electrochemical results indicate, the participation of GO sheets to the electrodeposition process, the GO sheets are expected to be found in the hybrid deposit. The morphology analysis shows that the addition of GO during electrodeposition of ZnO induces changes in morphology towards nanoplates, as well as in the growth direction from vertical to lateral, both at low and high cathodic potential, which could be attributed to the incorporation of GO sheets [56,57] where the oxygen groups in GO further react with Zn^2+^ ions [22]. As evidenced in the SEM images in Figure 4, Figure 5 and Figure 6, the nanostructures increase in terms of density and disorder with the GO content, and the morphology turns to concave nanostructures, suggesting that GO sheets are adsorbed on (001) planes of ZnO through their oxygen groups, resulting in stacks of platelets standing on the substrate, similarly to other reports on electrodeposition of ZnO in the presence of dye or surfactant molecules [58]. The nanocontainers obtained at a low potential of −0.8 V indicate an impeded growth of the polar facet of ZnO, which is associated with the presence of GO [48]. Thus, as long as the GO sheets only reversibly adsorb to the surface, they cannot be detected in the deposited films in certain potential and GO content conditions. Such a growth mechanism is further supported by the image of ZnO deposited at an even lower potential, depicted in Figure 6E where the ZnO morphology gets to concave spherical structures.

The XRD results indicate that the resulting ZnO possesses a high crystallinity [37], with a preferential growth orientation at a low GO content and high potential and loss of preferred orientation upon an increased GO content and at a low deposition potential. The results obtained indicate that the GO could be employed as a structure-directing agent for the electrodeposition of ZnO towards the fabrication of novel materials with synergetic properties.

## 5. Conclusions

ZnO nanostructures were obtained by electrochemical deposition. Graphene oxide was exploited as a structure-directing for ZnO by employing the GO as a surfactant during the electrodeposition of ZnO. Both the electrodeposition of ZnO and electro-reduction of GO take place simultaneously. The effect of deposition potential and duration on the morphology and structure of ZnO was analyzed. The Raman spectroscopy results indicate a successful modification of ZnO with GO sheets with the appearance of typical bands of both components. A hybridization threshold of 10 mg L^−1^ is indicated by the evolution of the defect-related band of ZnO at 580 cm^−1^. The morphology analysis shows that a low GO content preserves the morphology and orientation of ZnO nanostructures while a high content such as 10 mg L^−1^ changes the morphology in nanoplates and growth orientation to lateral. The results indicate that the approach is valid for the use of GO as a structure-directing agent for the fabrication of ZnO nanostructures by electrodeposition with varying morphologies and orientations.

## References

[1] Yang T., Chen M., Kong Q., Wang X., Guo X., Li W., Jiao K. (2015). Shape-controllable ZnO nanostructures based on synchronously electrochemically reduced graphene oxide and their morphology-dependent electrochemical performance. Electrochim. Acta.

[2] Rosas-Laverde N.M., Pruna A., Cembrero J., Orozco-Messana J., Manjón F.J. (2019). Performance of graphene oxide-modified electrodeposited ZnO/Cu_2_O heterojunction solar cells. Boletín Soc. Española Cerámica Y Vidr..

[3] Rosas-Laverde N.M., Pruna A., Busquets-Mataix D., Marí B., Cembrero J., Salas Vicente F., Orozco-Messana J. (2018). Improving the properties of Cu_2_O/ZnO heterojunction for photovoltaic application by graphene oxide. Ceram. Int..

[4] Zhang Z., Ren L., Han W., Meng L., Wei X., Qi X., Zhong J. (2015). One-pot electrodeposition synthesis of ZnO/graphene composite and its use as binder-free electrode for supercapacitor. Ceram. Int..

[5] Psychoyios V.N., Nikoleli G.-P.P., Tzamtzis N., Nikolelis D.P., Psaroudakis N., Danielsson B., Israr M.Q., Willander M. (2013). Potentiometric Cholesterol Biosensor Based on ZnO Nanowalls and Stabilized Polymerized Lipid Film. Electroanalysis.

[6] Jiang X., Lin Q., Zhang M., He G., Sun Z. (2015). Microstructure, optical properties, and catalytic performance of Cu_2_O-modified ZnO nanorods prepared by electrodeposition. Nanoscale Res. Lett..

[7] Cui J. (2012). Zinc oxide nanowires. Mater. Charact..

[8] Da Fonseca A.F.V., Siqueira R.L., Landers R., Ferrari J.L., Marana N.L., Sambrano J.R., de La Porta F.A., Schiavon M.A. (2018). A theoretical and experimental investigation of Eu-doped ZnO nanorods and its application on dye sensitized solar cells. J. Alloys Compd..

[9] Wang C., Wang Z.-G., Xi R., Zhang L., Zhang S.-H., Wang L.-J., Pan G.-B. (2019). In situ synthesis of flower-like ZnO on GaN using electrodeposition and its application as ethanol gas sensor at room temperature. Sens. Actuators B Chem..

[10] Marimuthu T., Anandhan N., Thangamuthu R., Surya S., Panneerselvam R., Ganesan K.P. (2019). Effect of Deposition Potential and Bath Temperature on One-Step Electrochemical Synthesis of One and Two Dimensional Nanostructured ZnO Thin Films on Fluorine Doped Tin Oxide Substrates. J. Nanosci. Nanotechnol..

[11] Rosas-Laverde N.M., Pruna A., Mele P. (2019). Electrodeposition of ZnO Nanostructured Films for Photovoltaics and Photoelectrochemical Sensing. ZnO Thin Films Properties, Performance and Applications.

[12] Al-Nassar S.I., Hussein F.I., Adel K.M. (2019). The effect of laser pulse energy on ZnO nanoparticles formation by liquid phase pulsed laser ablation. J. Mater. Res. Technol..

[13] Ozerov I., Bulgakov A.V., Nelson D.K., Castell R., Marine W. (2005). Production of gas phase zinc oxide nanoclusters by pulsed laser ablation. Appl. Surf. Sci..

[14] Shao Q., Chen S.Q.Y., Yeung O.L., Foo Y.S., Ng S.M., Zapien J.A., Leung C.W., Ruotolo A. (2016). Magnetism as a tool for band-gap narrowing of zinc oxide films prepared by sol–gel method. J. Sol Gel Sci. Technol..

[15] Wang X.L., Luan C.Y., Shao Q., Pruna A., Leung C.W., Lortz R., Zapien J.A., Ruotolo A. (2013). Effect of the magnetic order on the room-temperature band-gap of Mn-doped ZnO thin films. Appl. Phys. Lett..

[16] Haga K., Kamidaira M., Kashiwaba Y., Sekiguchi T., Watanabe H. (2000). ZnO thin films prepared by remote plasma-enhanced CVD method. J. Cryst. Growth.

[17] Abd Samad N.A., Lai C.W., Abd Hamid S.B. (2015). Easy Formation of Nanodisk-Dendritic ZnO Film via Controlled Electrodeposition Process. J. Nanomater..

[18] Künze S., Schlettwein D. (2014). Electrochemical and electroless deposition of porous zinc oxide on aluminium. Electrochim. Acta.

[19] Neuthe K., Bittner F., Stiemke F., Ziem B., Du J., Zellner M., Wark M., Schubert T., Haag R. (2014). Phosphonic acid anchored ruthenium complexes for ZnO-based dye-sensitized solar cells. Dyes Pigments.

[20] Ichinose K., Mizuno T., Schuette White M., Yoshida T. (2014). Control of Nanostructure and Crystallographic Orientation in Electrodeposited ZnO Thin Films via Structure Directing Agents. J. Electrochem. Soc..

[21] Nguyen T.H.Q., Ruess R., Schlettwein D. (2019). Adjusting Porosity and Pore Radius of Electrodeposited ZnO Photoanodes. J. Electrochem. Soc..

[22] Zou J.-P., Ma J., Huang Q., Luo S.-L., Yu J., Luo X.-B., Dai W.-L., Sun J., Guo G.-C., Au C.-T. (2014). Graphene oxide as structure-directing and morphology-controlling agent for the syntheses of heterostructured graphene-Bi_2_MoO_6_/Bi_3.64_Mo_0.36_O_6.55_ composites with high photocatalytic activity. Appl. Catal. B Environ..

[23] Wei A., Xiong L., Sun L., Liu Y., Li W., Lai W., Liu X., Wang L., Huang W., Dong X. (2013). One-step electrochemical synthesis of a graphene–ZnO hybrid for improved photocatalytic activity. Mater. Res. Bull..

[24] Ong W.-J., Tan L.-L., Chai S.-P., Yong S.-T. (2015). Graphene oxide as a structure-directing agent for the two-dimensional interface engineering of sandwich-like graphene-G-C_3_ N_4_ hybrid nanostructures with enhanced visible-light photoreduction of CO_2_ to methane. Chem. Commun..

[25] Hummers W.S., Offeman R.E. (1957). Preparation of Graphitic Oxide. J. Am. Chem. Soc..

[26] Du Y., Yao H., Zhao L., Yang H., Wang M., Yuan L., Xu Y., Li J. (2019). Graphene Oxide Induced High Crystallinity of SAPO-11 Molecular Sieves for Improved Alkane Isomerization Performance. ChemNanoMat.

[27] Liu C., Liu H., Xiong T., Xu A., Pan B., Tang K. (2018). Graphene Oxide Reinforced Alginate/PVA Double Network Hydrogels for Efficient Dye Removal. Polymer.

[28] Muthu Prabhu S., Park C.M., Shahzad A., Lee D.S. (2019). Designed synthesis of sulfide-rich bimetallic-assembled graphene oxide sheets as flexible materials and self-tuning adsorption cum oxidation mechanisms of arsenic from water. J. Mater. Chem. A.

[29] Yang J., Hao J., Xu S., Dai J., Wang Y., Pang X. (2018). Visible-light-driven photocatalytic degradation of 4-CP and the synergistic reduction of Cr(VI) on one-pot synthesized amorphous Nb_2_O_5_ nanorods/graphene heterostructured composites. Chem. Eng. J..

[30] Li F., Xie L., Sun G., Kong Q., Su F., Lei H., Guo X., Zhang B., Chen C. (2017). Regulating pore structure of carbon aerogels by graphene oxide as ‘shape-directing’ agent. Microporous Mesoporous Mater..

[31] Zhang X., Fan Q., Qu N., Yang H., Wang M., Liu A., Yang J. (2019). Ultrathin 2D nitrogen-doped carbon nanosheets for high performance supercapacitors: Insight into the effects of graphene oxides. Nanoscale.

[32] Zhang M., Wu M., Liu Q., Wang X., Lv T., Jia L. (2017). Graphene oxide mediated cellulose-derived carbon as a highly selective catalyst for the hydrolysis of cellulose to glucose. Appl. Catal. A Gen..

[33] Cai J., Lu J.-Y., Chen Q.-Y., Qu L.-L., Lu Y.-Q., Gao G.-F. (2017). Eu-Based MOF/graphene oxide composite: A novel photocatalyst for the oxidation of benzyl alcohol using water as oxygen source. New J. Chem..

[34] Zhang L., He W., Ling M., Shen K., Liu Y., Guo S. (2017). Self-standing MgMoO_4_/Reduced Graphene Oxide Nanosheet Arrays for Lithium and Sodium Ion Storage. Electrochim. Acta.

[35] Lu Y., Jiang Y., Chen W. (2014). Graphene nanosheet-tailored PtPd concave nanocubes with enhanced electrocatalytic activity and durability for methanol oxidation. Nanoscale.

[36] Teh S.J., Yeoh S.L., Lee K.M., Lai C.W., Abdul Hamid S.B., Thong K.L. (2016). Effect of reduced graphene oxide-hybridized ZnO thin films on the photoinactivation of Staphylococcus aureus and Salmonella enterica serovar Typhi. J. Photochem. Photobiol. B Biol..

[37] Pruna A., Wu Z., Zapien J.A.A., Li Y.Y.Y., Ruotolo A. (2018). Enhanced photocatalytic performance of ZnO nanostructures by electrochemical hybridization with graphene oxide. Appl. Surf. Sci..

[38] Bu Y., Chen Z., Li W., Hou B. (2013). Highly Efficient Photocatalytic Performance of Graphene–ZnO Quasi-Shell–Core Composite Material. ACS Appl. Mater. Interfaces.

[39] Pruna A., Cembrero J., Pullini D., Mocioiu A.M., Busquets-Mataix D. (2017). Effect of reduced graphene oxide on photocatalytic properties of electrodeposited ZnO. Appl. Phys. A.

[40] Maiti S., Pal S., Chattopadhyay K.K. (2015). Recent advances in low temperature, solution processed morphology tailored ZnO nanoarchitectures for electron emission and photocatalysis applications. CrystEngComm.

[41] Pruna A., Pullini D., Busquets D. (2015). Structure and Properties of Chemically-reduced Functionalized Graphene Oxide Platelets. J. Mater. Sci. Technol..

[42] Zhou M., Wang Y., Zhai Y., Zhai J., Ren W., Wang F., Dong S. (2009). Controlled Synthesis of Large-Area and Patterned Electrochemically Reduced Graphene Oxide Films. Chem. A Eur. J..

[43] Septina W., Ikeda S., Khan M.A., Hirai T., Harada T., Matsumura M., Peter L.M. (2011). Potentiostatic electrodeposition of cuprous oxide thin films for photovoltaic applications. Electrochim. Acta.

[44] Wu H., Zhao X., Li J., Dong S. (2017). The large-area preparation and photoelectrochemical properties of graphene/ZnO nanorod composite film. RSC Adv..

[45] Sánchez Zeferino R., Barboza Flores M., Pal U. (2011). Photoluminescence and raman scattering in ag-doped zno nanoparticles. J. Appl. Phys..

[46] Wu S., Yin Z., He Q., Huang X., Zhou X., Zhang H. (2010). Electrochemical deposition of semiconductor oxides on reduced graphene oxide-based flexible, transparent, and conductive electrodes. J. Phys. Chem. C.

[47] Wang Y., Wang F., He J. (2013). Controlled fabrication and photocatalytic properties of a three-dimensional ZnO nanowire/reduced graphene oxide/CdS heterostructure on carbon cloth. Nanoscale.

[48] Pan X., Yang M.Q., Xu Y.J. (2014). Morphology control, defect engineering and photoactivity tuning of ZnO crystals by graphene oxide—A unique 2D macromolecular surfactant. Phys. Chem. Chem. Phys..

[49] Skompska M., Zarȩbska K. (2014). Electrodeposition of ZnO nanorod arrays on transparent conducting substrates—A review. Electrochim. Acta.

[50] Arslan A., Hür E., Ilican S., Caglar Y., Caglar M. (2014). Controlled growth of c-axis oriented ZnO nanorod array films by electrodeposition method and characterization. Spectrochim. Acta Part A Mol. Biomol. Spectrosc..

[51] Zhou Y., Li D., Yang L., Li C., Liu Y., Lu J., Wang Y. (2017). Preparation of 3D urchin-like RGO/ZnO and its photocatalytic activity. J. Mater. Sci. Mater. Electron..

[52] Li Y., Wang D., Li W., He Y. (2015). Photoelectric conversion properties of electrochemically codeposited graphene oxide–ZnO nanocomposite films. J. Alloys Compd..

[53] Ambrosi A., Pumera M. (2013). Precise tuning of surface composition and electron-transfer properties of graphene oxide films through electroreduction. Chem. A Eur. J..

[54] Gutić S.J., Kozlica D.K., Korać F., Bajuk-Bogdanović D., Mitrić M., Mirsky V.M., Mentus S.V., Pašti I.A. (2018). Electrochemical tuning of capacitive response of graphene oxide. Phys. Chem. Chem. Phys..

[55] Viinikanoja A., Wang Z., Kauppila J., Kvarnström C. (2012). Electrochemical reduction of graphene oxide and its in situ spectroelectrochemical characterization. Phys. Chem. Chem. Phys..

[56] Moshgi Asl S., Afshar A., Yaghoubinezhad Y. (2018). An Electrochemical Synthesis of Reduced Graphene Oxide/Zinc Nanocomposite Coating through Pulse-Potential Electrodeposition Technique and the Consequent Corrosion Resistance. Int. J. Corros..

[57] Henni A., Harfouche N., Karar A., Zerrouki D., Perrin F.X., Rosei F. (2019). Synthesis of graphene–ZnO nanocomposites by a one-step electrochemical deposition for efficient photocatalytic degradation of organic pollutant. Solid State Sci..

[58] Oekermann T., Yoshida T., Schlettwein D., Sugiura T., Minoura H. (2001). Photoelectrochemical properties of ZnO/tetrasulfophthalocyanine hybrid thin films prepared by electrochemical self assembly. Phys. Chem. Chem. Phys..

